# 817. A 4-Year Retrospective Review on a Burn Center’s Experience with a Collagen-elastin Matrix

**DOI:** 10.1093/jbcr/irag033.256

**Published:** 2026-04-06

**Authors:** Tait Olaveson

**Affiliations:** The Burn Center at Eastern Idaho Regional Medical Center, Rigby, ID

## Abstract

**Introduction:**

A collagen-elastin matrix (CEM) has recently been approved for clinical use in the United States after more than 25 years of international application. Adoption of CEM in our rural practice has altered reconstructive strategies, particularly by reducing time to definitive closure in both burn and wound patients.

**Methods:**

We retrospectively reviewed cases treated with CEM between January 2022 and September 2025. Cases were stratified by age of patient, denoting pediatric (< 18 years) and adult groups, as well as type of injury, specifically burns versus wounds. Burn patients were further categorized by injury extent (< 15% vs. >15% TBSA). Additional data points include time from application of CEM to definitive closure and frequency of simultaneous split-thickness skin grafting (STSG) with CEM placement.

**Results:**

From January 2022 to September 2025, there were a total of 202 cases with CEM usage. Of these cases, 186 (92%) adults and 16 (8%) pediatric patients were treated with CEM. When looking at type of injury, 70 (34.7%) sustained burns, and 132 (65.3%) had wound-related diagnoses. Of the burn cases, 44 (63%) were minor burns (≤15% TBSA) and 26 (37%) were major burns (>15% TBSA). All of the burn cases involved 3rd degree burns. Early in our experience, time from CEM application to definitive closure averaged 12 days, consistent with prior dermal matrix use. With increasing experience, this interval decreased to 8 days overall. Among wound patients, time to closure decreased from 12 to 9.3 days, while burn patients demonstrated a reduction from 12 to 6.9 days. Additionally, 51 (25.2%) of all patients underwent simultaneous CEM placement and STSG during a single operative session.

**Conclusions:**

Use of CEM in our rural practice has resulted in significant reductions in time to definitive closure, particularly among wound patients, with parallel improvements in burn management. These findings highlight the evolving role of CEM in expediting reconstructive outcomes.

**Applicability of Research to Practice:**

Decreased time to closure translates to reduced hospital length of stay and faster return to activities of daily living (ADLs). CEM provides a reproducible option for optimizing closure strategies in both pediatric and adult patients in rural and resource-limited settings.

**Funding for the study:**

N/A.

